# Promoting Flexibility and Functionality in a Surgically Managed Tibial Fracture: A Case Report on Physiotherapeutic Interventions for Postoperative Stiffness

**DOI:** 10.7759/cureus.50589

**Published:** 2023-12-15

**Authors:** Krishnayani Shende, Grisha Ratnani, Nishigandha P Deodhe, Khushi M Gandhi

**Affiliations:** 1 Neurophysiotherapy, Ravi Nair Physiotherapy College, Datta Meghe Institute of Higher Education and Research, Wardha, IND

**Keywords:** osteosynthesis plate physical therapy, rehabilitation, open reduction and internal fixation, tibia fracture, mulligan mobilization

## Abstract

This case study explains the complete care of a 45-year-old male patient who had a high-impact road injury that resulted in a displaced proximal tibial fracture. Substantial soft tissue damage was discovered during the initial assessment, requiring careful thought before undergoing surgery. A customized physiotherapy program was instituted after an incremental strategy involving open reduction and internal fixation. The patient made a satisfactory functional recovery, regaining nearly normal mobility and going back to daily activities within 12 weeks despite difficulties encountered during the rehabilitation phase, including temporary postoperative complications. The present study underscores the significance of a multidisciplinary approach involving Mulligan mobilization in the effective management of intricate proximal tibial fractures. It also underscores the importance of meticulous surgical intervention and organized rehabilitation protocols in enhancing patient outcomes and regaining functional abilities to improve patients' quality of life.

## Introduction

A tibial fracture is a common and serious lower extremity injury that is characterized by a break or crack in the shinbone. These fractures are frequently caused by high-energy trauma, but they can also result from overuse or underlying illnesses that weaken the bone [1]. The age, sex, location, and lifestyle of an individual can all have an impact on the incidence of tibial fractures. Tibial fractures are among the most common long bone fractures, accounting for 10-15% of all adult fractures [2]. An estimated 492,000 visits per year were attributed to tibial fractures in the United States alone [3]. Since these fractures can seriously impair a person's mobility, prompt and appropriate care is frequently required for the best possible healing functional recovery and quality of life [4]. According to the American Academy of Orthopaedic Surgeons, tibial fracture accounts for 85% of all lower extremity fractures, making them an important part of orthopaedic medicine. Because of the inherent instability and limited potential for bone healing, multiple bone fragments in a fracture, also referred to as a comminuted fracture, present additional challenges [5]. Traumatic tibial fractures can take many different forms and range in severity, rendering individuals immobile and requiring immediate medical care. Conservative measures and surgical interventions, such as internal or external fixation, are among the available treatment options [1].

Intense lower limb pain is one of the symptoms and indicators, and it frequently gets worse with movement or activities that require bearing weight on the affected limbs. Together with a visible deformity or angulation of the leg, there may also be pain and swelling near the fracture site. The afflicted limb might be unable to support much weight, and the area around the wound might be bruised or discoloured. A complex fracture may, in extreme circumstances, result in an open wound or bone protrusion. In more severe cases, tingling or numbness may also be caused by damaged blood vessels or nerve involvement [6].

Open reduction and internal fixation (ORIF) is a common surgical procedure used to stabilize and align fractured bones, particularly tibial plateau fractures, with the use of a plate and screws. For the treatment of proximal tibial fractures involving the tibial plateau, especially those that are displaced or comminuted, ORIF using a plate and screws is commonly used. Using an open reduction technique, the fractured bone is exposed, the fragments are realigned, and then they are stabilized with a metal plate and screws. In order to guarantee the best possible long-term function, the main objectives of this procedure are to promote early mobilization, improve fracture healing, and restore the tibia's articular integrity. The strength and stability of the fixation have been significantly improved by the use of locking screws and anatomically shaped plates, which has decreased the incidence of non-union and malunion in tibial fracture cases. To reduce the risk of complications like infection, implant failure, and delayed wound healing, it is essential to carefully evaluate the soft tissue condition, fracture pattern, and patient-specific factors [7].

Plate osteosynthesis, internal fixation, and open reduction are frequently used in the treatment of tibial fractures and have shown promise in improving functional recovery and enabling early weight bearing. This is especially noticeable in cases of complex or extremely unstable fractures. Strict adherence to well-defined rehabilitation protocols and close patient monitoring after surgery is essential for optimizing the overall recovery process and reducing the risk of complications [8]. Musculoskeletal disorders that are known to be difficult to treat, like complicated De Quervain's syndrome and lateral epicondylitis, are generally not treated with manual therapy. On the other hand, Mulligan's mobilization with movement (MWM)-based therapeutic approaches are becoming more well-known for their efficacy in these circumstances. In manual therapy treatment, a specific joint glide is maintained during a compromised function by applying a manual force in MWM. The impaired joint can move freely, pain-free, with this technique called MWM. In MWM, the force is typically applied perpendicular to the plane of movement or impaired action and parallel to the treatment plane. We do not yet know the precise mechanisms by which MWM enhances patient outcomes. MWM may be able to correct joint positional errors brought on by sprains or injuries. To completely comprehend this suggested mechanism of action, more study is necessary, though, especially regarding the controversy surrounding other spinal manipulation theories like chiropractic subluxation [9].

The significance of customizing treatment regimens to meet each patient's unique needs is emphasized by this case study. It describes a multidisciplinary strategy that was used to treat stiffness after a tibia fracture that had comminuted. There are various benefits to using Mulligan mobilization to treat knee stiffness. This method uses mild and painless mobilization techniques during targeted movements in an effort to improve joint mechanics, lessen pain, and improve functional outcomes [10]. Unlike conventional methods, Mulligan mobilization actively involves patients in their rehabilitation process. Better results and higher patient compliance are frequently the results of this proactive involvement [11].

## Case presentation

A 45-year-old male patient reported he had been experiencing pain in his left knee joint over three months. He also reported stiffness in the same joint and difficulty walking. Three months prior, the patient underwent surgical management for a tibial fracture that was treated with ORIF. After seeing an orthopaedic department, the patient was given medication prescriptions and directed to the physiotherapy department for additional care. Table 1 presents the current episodes' chronology.

**Table 1:** Timeline of current episodes

Events	Dates
Date of accident	July 03, 2023
Date of operation	July 10, 2023
Duration of physical therapy rehabilitation	Three weeks (October 21 to November 10, 2023)

Clinical findings

The patient was conscious, cooperative, and able to follow instructions and had a mesomorphic build. When the patient was first observed, the head was raised at a 30-degree angle while the patient was reclining in a supine position. While the patient was in the supine position, his posture was assessed. The ankle was in a plantarflexed position, the knee was flexed, and the left hip was extended. Upon examination of the left leg, the skin covering appeared tense, and there was generalized swelling observed over the upper portion of the leg and knee. A bandage was present on the anterior aspect of the left knee. Upon palpation, the local temperature was within normal limits. Tenderness was detected over the upper portion of the left leg, specifically over the tibial condyles. The dimensions of the bandage were measured to be 15 cm x 6 cm. The range of motion (ROM) at the knee joint is documented in Table 2.

**Table 2:** The range of motion of the knee joint

Knee joint	Right	Left
Flexion	0-120^o^	0-30^o^
Extension	120-0^o^	30-0^o^

Clinical investigations

**Figure 1:**
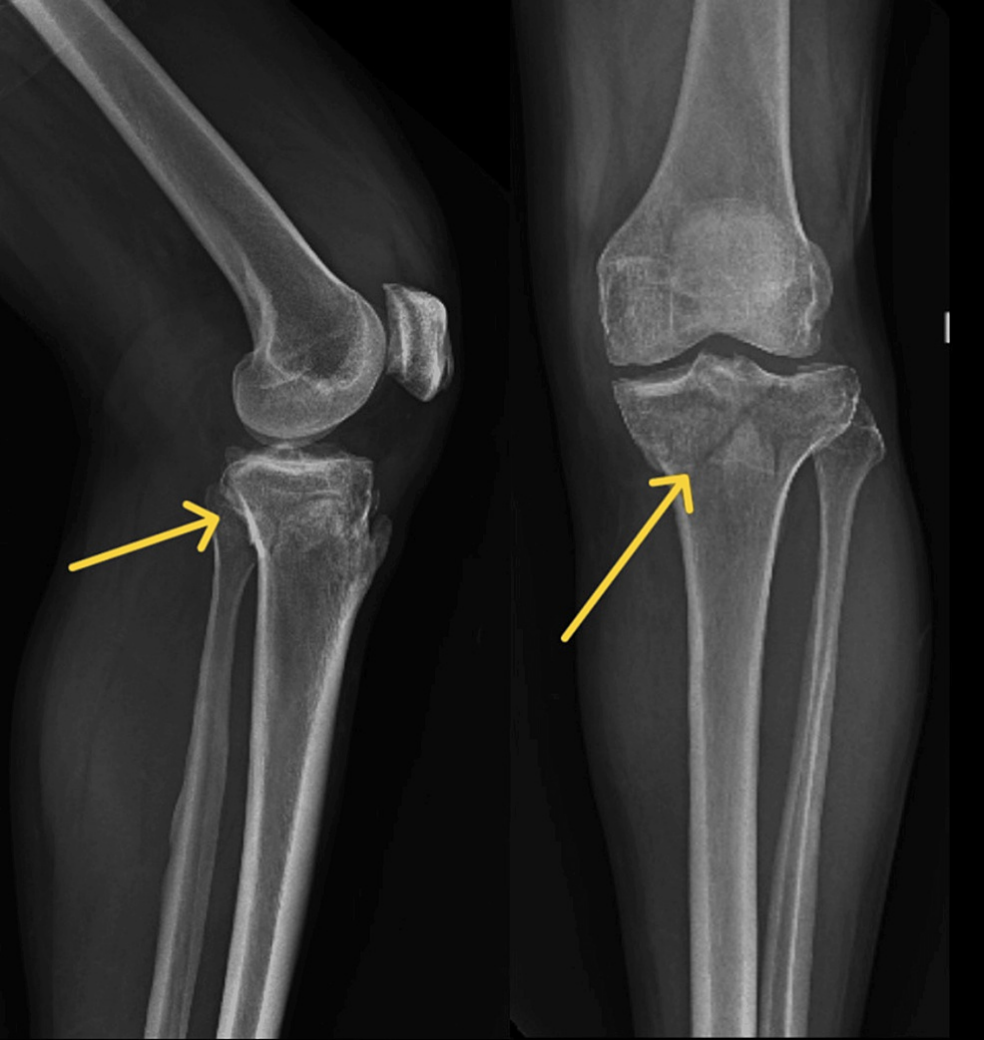
The pre-operative radiograph of the left knee joint lateral and anteroposterior views

**Figure 2:**
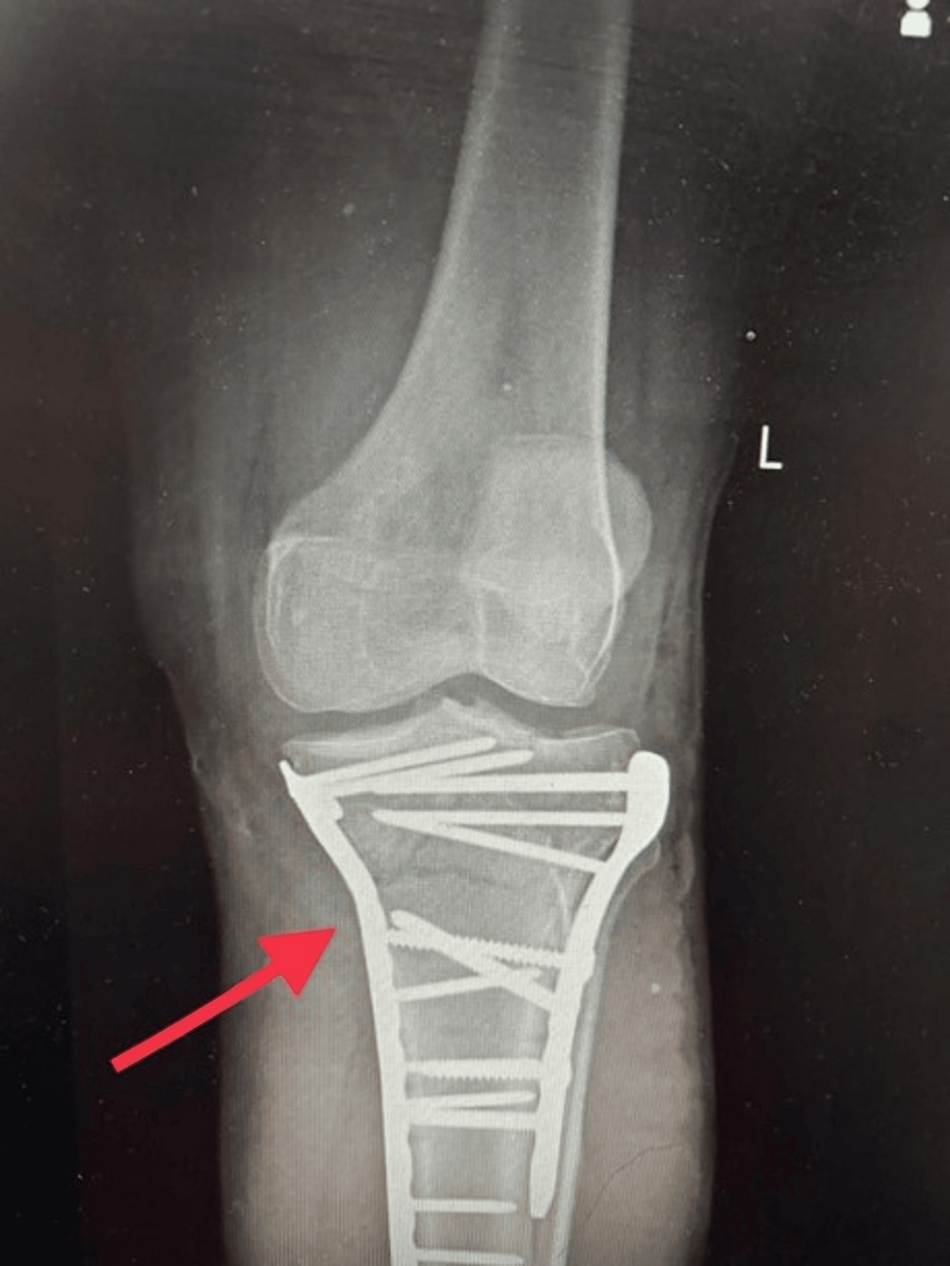
The post-operative radiograph of the left knee joint fixed with screws and plates

**Figure 3:**
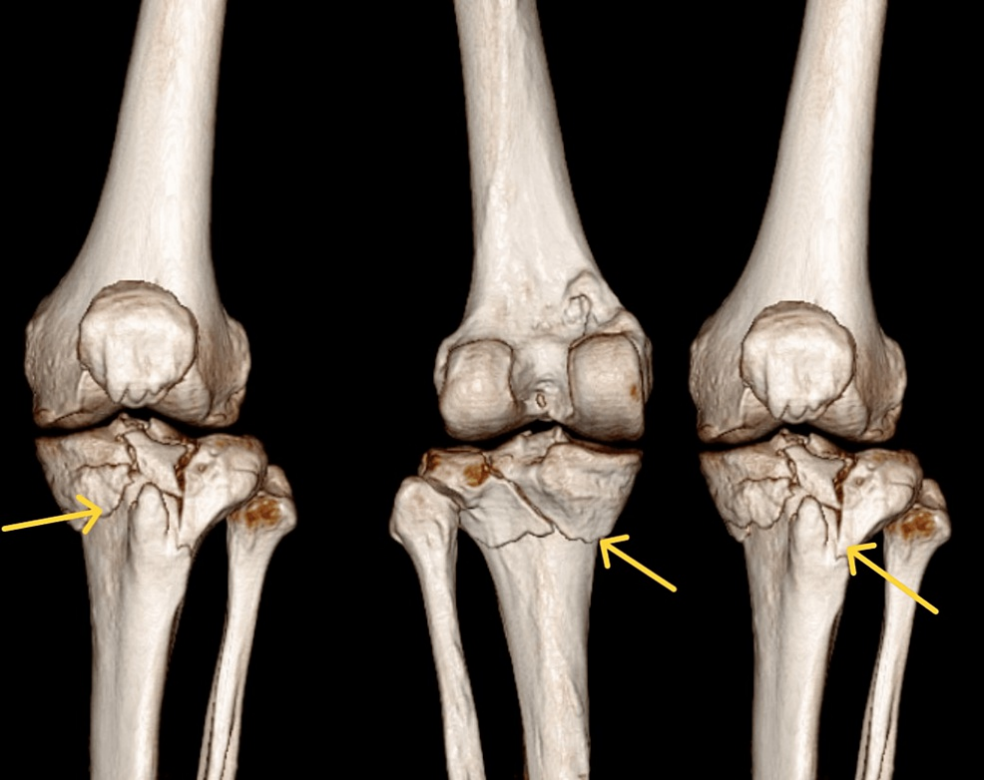
CT scan of the left knee joint

Figure 1 depicts a pre-operative X-ray. A wedge fracture of the medial and lateral tibial plateau is shown on preoperative radiographs, suggesting a type V Schatzker classification. Figure 2 shows the X-ray taken after surgery. The radiograph in Figure 2 illustrates how screws and plates are used to fix a type V Schatzker fracture internally. CT scan seen in Figure 3 demonstrates comminuted bicondylar fracture of the tibia with tibial eminence fracture that is Schatzker classification type V.

Treatment

Physiotherapy Intervention

The physiotherapeutic intervention lasted for a duration of three weeks, with a frequency of five sessions per week, each session lasting approximately 40 minutes.

Week 1: In order to relieve pain, the patient was advised to have cryotherapy sessions thrice daily for 10 minutes focused on the left knee joint. In order to avoid oedema and lessen swelling, it was also advised that the patient elevate the afflicted limb. Gentle passive stretching exercises were given, with three 30-second holds, to improve quadriceps flexibility. In order to strengthen the glutes, hamstrings, and quadriceps, isometric exercises were also recommended. It was suggested to perform straight leg raises to increase general strength. To improve mobility, active ROM exercises were recommended. Additionally, it was advised to perform the vastus medialis oblique (VMO) strengthening exercise in a single set of 10 repetitions. Mulligan mobilization for the knee joint was administered, which entails ten separate sets of MWM, to ease pain and reduce stiffness.

Week 2: The patient was instructed to perform three sets of light passive stretching, with a 30-second rest in between, in order to increase the flexibility of their quadriceps. Furthermore, perform isometric workouts targeting the quadriceps, hamstrings, and glutes. Leg raises done straight are one type of strengthening exercise. Active ROM exercises were done to increase mobility. He did dynamic squats and mini squats, two weight-bearing exercises, 10 times. In addition, there were three sets of 10 repetitions of VMO strengthening and Mulligan mobilization for the knee joint. To ease pain and lessen stiffness, MWM was employed for ten distinct sets in addition to proprioceptive training. It was advised to ice for 10 minutes three times a day.

Week 3:* *To improve the strength of the quadricep muscles, a dynamic training program was employed, which involved performing ten repetitions in a single set with a one-kilogram weight. Furthermore, two sets of 10 repetitions of VMO strength training exercises were performed with resistance. Mulligan mobilization was applied to the knee joint, involving 15 separate sets. The quadriceps were further strengthened with 20 mini squats. Ten repetitions of the muscle energy technique were used to increase the muscle strength. Lastly, icing the affected area three times a day for 10 minutes each time can help with relief and recovery.

**Figure 4:**
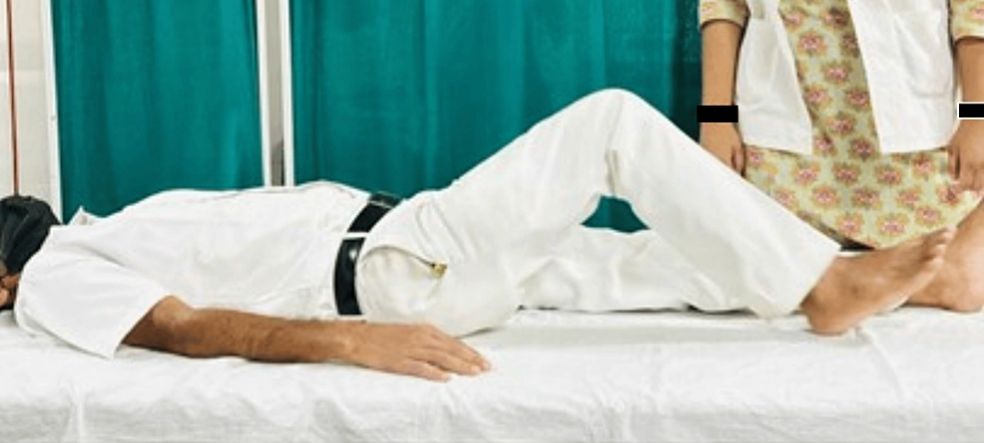
The patient performing active range of motion exercises.

**Figure 5:**
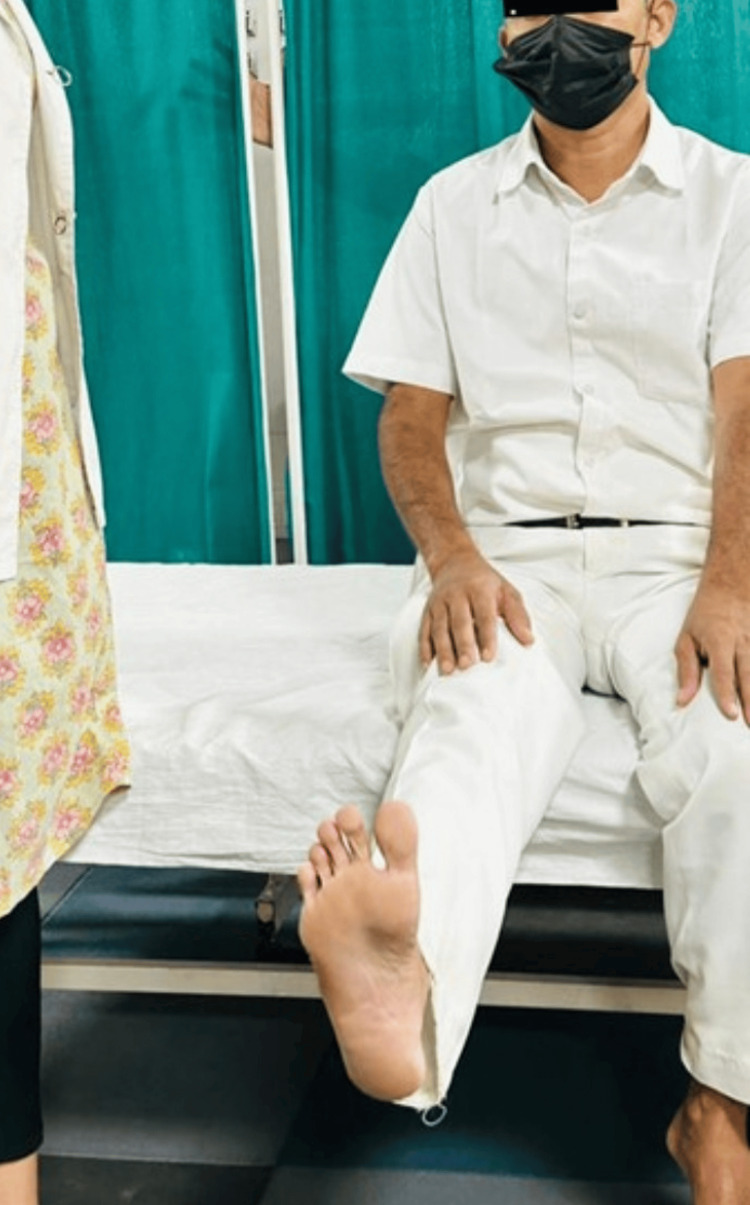
The patient performing dynamic quads.

Physiotherapists and other medical professionals use Mulligan mobilization, a manual therapy technique, to treat musculoskeletal pain and movement limitations [12]. This approach combines prolonged joint mobilization with active patient mobility. The main objectives are to improve joint mobility and relieve pain during particular movements [10]. On the other hand, traditional methods include the standard physiotherapy exercises and techniques used to treat musculoskeletal problems. To increase strength, flexibility, and general function, these methods could involve massage, stretches, exercises, and other modalities [13]. Figures 4-5 show patients performing exercises.

Follow-up and outcomes

An interventional physical therapy process was initiated after thorough planning. A follow-up was performed once a week for four weeks. Telerehabilitation was used for routine post-discharge follow-up. Outcome measures are listed in Tables 3-4.

**Table 3:** Outcome measures before and after the treatment

Outcome measures	On admission	On discharge
Numerical pain rating scale	9/10	4/10
Lower extremity functional scale	7/80	36/80

**Table 4:** Before and after the assessment of range of motion

Joint	Movement	On admission	At discharge	On admission	At discharged
		Left	Left	Right	Right
Hip	Extension	0-70	0-90	0-130	0-130
	Flexion	0-15	0-25	0-30	0-30
Knee	Extension	0-10	0-60	0-120	0-120
	Flexion	10-0	0-60	0-120	0-120
Ankle	Plantar-flexion	0-50	0-60	0-40	0-40
	Dorsiflexion	0-16	0-25	0-25	0-25

## Discussion

This case study features a 45-year-old male patient who had a proximal tibial fracture managed surgically with ORIF. After three months, the patient visited the orthopaedic department for follow-up with complaints of stiffness, pain and restricted ROM, for the same he was referred to physical therapy. Preventing subsequent problems, enhancing lower limb strength and ROM, reducing discomfort, and encouraging early weight-bearing were the main objectives of physiotherapy management. An extensive review of pertinent literature is conducted to determine the efficacy of Mulligan MWM approaches in the treatment of osteoarthritis in the knee [14]. Changes in knee ROM, pain alleviation, and improved function are just a few of the outcomes that are included in the evaluation. In addition, a randomized clinical trial is being conducted to evaluate and compare Mulligan's mobilization techniques and ischemia compression as treatments for patellofemoral pain syndrome. In order to identify the most efficient method for treating this condition with manual therapy approaches, the study focuses on knee function, pain severity, and patient-reported outcomes [15].

According to Gaston et al., 20% of patients with tibial plateau fractures experience stiffening (a persistent knee flexion contracture of greater than 5º) a year following surgery [16]. Flexion contractures, extension contractures, and combination contractures are the three categories into which Pujol et al. have classified the causes of post-traumatic knee stiffness. Fibrotic alteration of periarticular tissues and/or extensive intra-articular adhesions may be the cause of post-traumatic stiffness. In general, posterior adhesions and/or anterior impingement are the cause of flexion contractures. Conversely, posterior impingement or anterior adhesions cause extension contractures [17].

A comprehensive method of fracture care management emphasizes the significance of comprehending the biomechanics of tibial fractures. It offers justification for surgical fracture therapy and assesses the effects of different fixation techniques, such as intramedullary nailing, plate osteosynthesis, and external fixation, on soft tissue injuries and fracture patterns [18]. Tibial fracture-specific problems can be effectively addressed with Mulligan mobilization techniques. These techniques can address a variety of problems, such as the improvement of weight-bearing capacity, the restoration of normal gait patterns, and the correction of muscular imbalances that frequently result from immobilization. Following a fracture, patients can achieve a more thorough healing process, functional independence, and improved quality of life by concentrating on these areas [19]. According to Bhandari et al., two major advantages of operational treatment are enhanced fracture stability and early mobilization; the fact that our patient recovered well after surgery is evidence of the efficacy of these approaches [20].

## Conclusions

The results of this case study on physiotherapeutic therapies for postoperative stiffness in tibial fractures treated surgically demonstrate the important role that specialized physiotherapy plays in fostering functioning and flexibility. The case's comprehensive physiotherapy approach addressed postoperative stiffness in an effective manner, which increased the ROM and functional recovery. Tibial fractures require not just precise surgery but also an individualized approach to postoperative therapy for optimal care. This highlights the necessity of tailored approaches to reduce postoperative stiffness and improve the overall functional outcome, underscoring the significance of including physiotherapeutic therapies in the overall treatment strategy for tibial fractures. In order to achieve the best results, the case report highlights the benefits of working together between surgical treatments and focused physical therapy.
